# Impact of annual trend volume of low-dose computed tomography for lung cancer screening on overdiagnosis, overmanagement, and gender disparities

**DOI:** 10.1186/s40644-024-00716-5

**Published:** 2024-06-12

**Authors:** Chen Hsin-Hung, Tang En-Kuei, Wu Yun-Ju, Wu Fu-Zong

**Affiliations:** 1 Department of Medical Education and Research, Kaohsiung Veterans General Hospital, Kaohsiung, 813414 Taiwan; 2 Department of Surgery, Kaohsiung Veterans General Hospital, Kaohsiung, 813414 Taiwan; 3 Department of Radiology, Kaohsiung Veterans General Hospital, Kaohsiung, Taiwan; 4 Faculty of Medicine, School of Medicine, National Yang Ming Chiao Tung University, Taipei, Taiwan; 5 Faculty of Clinical Medicine, National Yang Ming Chiao Tung University, Taipei, Taiwan

**Keywords:** Overdiagnosis, Volume trend, Low-dose computed tomography

## Abstract

**Background:**

With the increasing prevalence of nonsmoking-related lung cancer in Asia, Asian countries have increasingly adopted low-dose computed tomography (LDCT) for lung cancer screening, particularly in private screening programs. This study examined how annual LDCT volume affects lung cancer stage distribution, overdiagnosis, and gender disparities using a hospital-based lung cancer database.

**Methods:**

This study analyzed the annual utilized LDCT volume, clinical characteristics of lung cancer, stage shift distribution, and potential overdiagnosis. At the individual level, this study also investigated the relationship between stage 0 lung cancer (potential strict definition regarding overdiagnosis) and the clinical characteristics of lung cancer.

**Results:**

This study reviewed the annual trend of 4971 confirmed lung cancer cases from 2008 to 2021 and conducted a link analysis with an LDCT imaging examination database over these years. As the volume of lung cancer screenings has increased over the years, the number and proportion of stage 0 lung cancers have increased proportionally.

Our study revealed that the incidence of stage 0 lung cancer increased with increasing LDCT scan volume, particularly during the peak growth period from 2017 to 2020. Conversely, stage 4 lung cancer cases remained consistent across different time intervals. Furthermore, the increase in the lung cancer screening volume had a more pronounced effect on the increase in stage 0 lung cancer cases among females than it had among males. The estimated potential for overdiagnosis brought about by the screening process, compared to non-participating individuals, ranged from an odds ratio of 7.617 to one of 17.114. Both strict and lenient definitions of overdiagnosis (evaluating cases of stage 0 lung cancer and stages 0 to 1 lung cancer) were employed.

**Conclusions:**

These results provide population-level evidence of potential lung cancer overdiagnosis in the Taiwanese population due to the growing use of LDCT screening, particularly concerning the strict definition of stage 0 lung cancer. The impact was greater in the female population than in the male population, especially among females younger than 40 years.

To improve lung cancer screening in Asian populations, creating risk-based prediction models for smokers and nonsmokers, along with gender-specific strategies, is vital for ensuring survival benefits and minimizing overdiagnosis.

**Supplementary Information:**

The online version contains supplementary material available at 10.1186/s40644-024-00716-5.

## Introduction

Lung cancer is the leading cause of cancer-related fatalities globally [1]. Recent research, encompassing clinical trials and meta-analyses, has demonstrated the efficacy of using low-dose computed tomography (LDCT) for lung cancer in significantly diminishing lung cancer mortality rates [2]. In light of evidence from European and American countries that demonstrates reduced lung cancer mortality rates among heavy smokers, Asian countries such as China, Japan, South Korea, and Taiwan have also adopted LDCT screening for both smoking and nonsmoking high-risk populations [3–5]. This is particularly relevant given the high prevalence of lung cancer among nonsmokers in Asian nations [6, 7]. However, emerging evidence shows the widespread real-world implementation of LDCT in smokers and nonsmokers in Asia has led to a rapid increase in first-stage lung cancer, raising potential concerns about overdiagnosis and the subsequent risk of overtreatment [8–10].

To date, no study has investigated the potential long-term consequences of widespread LDCT screening on the overdiagnosis of stage 0 lung cancer. The rationale of the current study was to explore how trends in LDCT volume over the years has impacted the potential overdiagnosis of stage 0 lung cancer in a hospital-based lung cancer registry. Therefore, the primary objective of this study was to investigate how the screening volume of indiscriminate and risk-based lung cancer screening conducted in the real world from 2008 to 2021 impacted the long-term trend changes in lung cancer registration data at a single hospital.

This investigation stemmed from the coexistence of self-paid and clinical trial-based lung cancer screening for both smoking and nonsmoking patients at varying risk levels in the real world. We collectively term this indiscriminate and risk-targeting screening, potentially altering lung cancer stage distribution. We examined how gender, age range, and different time periods (2009–2012, 2013–2016, and 2017–2020) affected potential overdiagnosis, manifested by stage 0 lung cancer. Considering variables like gender, age, smoking, betel nut and alcohol use, and screening status provides insights into addressing overdiagnosis. Analyzing clinical factors offers solutions for overdiagnosis. Stage 0 indicates pre-invasive lesions, while stage I denotes localized disease. Recognizing these stages guides treatment and surveillance decisions. Refining stage 0 to 1 lung cancer classification criteria is vital for assessing overdiagnosis magnitude across a spectrum of definitions.

## Methods

### Hospital-based lung cancer registry cohort

Since 2007, self-paid LDCT lung cancer screening at Kaohsiung Veterans General Hospital has been offered to individuals, mainly targeting those aged 40 to 80 years. This initiative was launched in response to the rising prevalence of nonsmoking-related lung cancer, driven by concerns about family history-related lung cancer, and screening promotions targeting nonsmoking-related lung cancer [6, 11]. We compiled data on the total number of LDCT screenings performed at our hospital between 2008 and 2021 using a database system of medical images. In this retrospective analysis, all LDCT exams adhered to the standard protocol for CT equipment and image acquisition settings. Radiologists conducted the report readings without AI software assistance. Furthermore, we acquired data on the temporal patterns of lung cancer incidence, mortality rates, stage distribution (including stage 0 lung cancer), death events, and lifestyle habits from our hospital’s lung cancer registry database that encompassed the same timeframe, 2008 to 2021. This investigation followed the guidelines set forth in the Declaration of Helsinki (revised in 2013) and received approval from the Institutional Review Board of Kaohsiung Veterans General Hospital (IRB number: KSVGH23-CT6-27). Given the retrospective nature of the study, the necessity for patient consent was exempted.

### Study design and flowchart

**Fig. 1:**
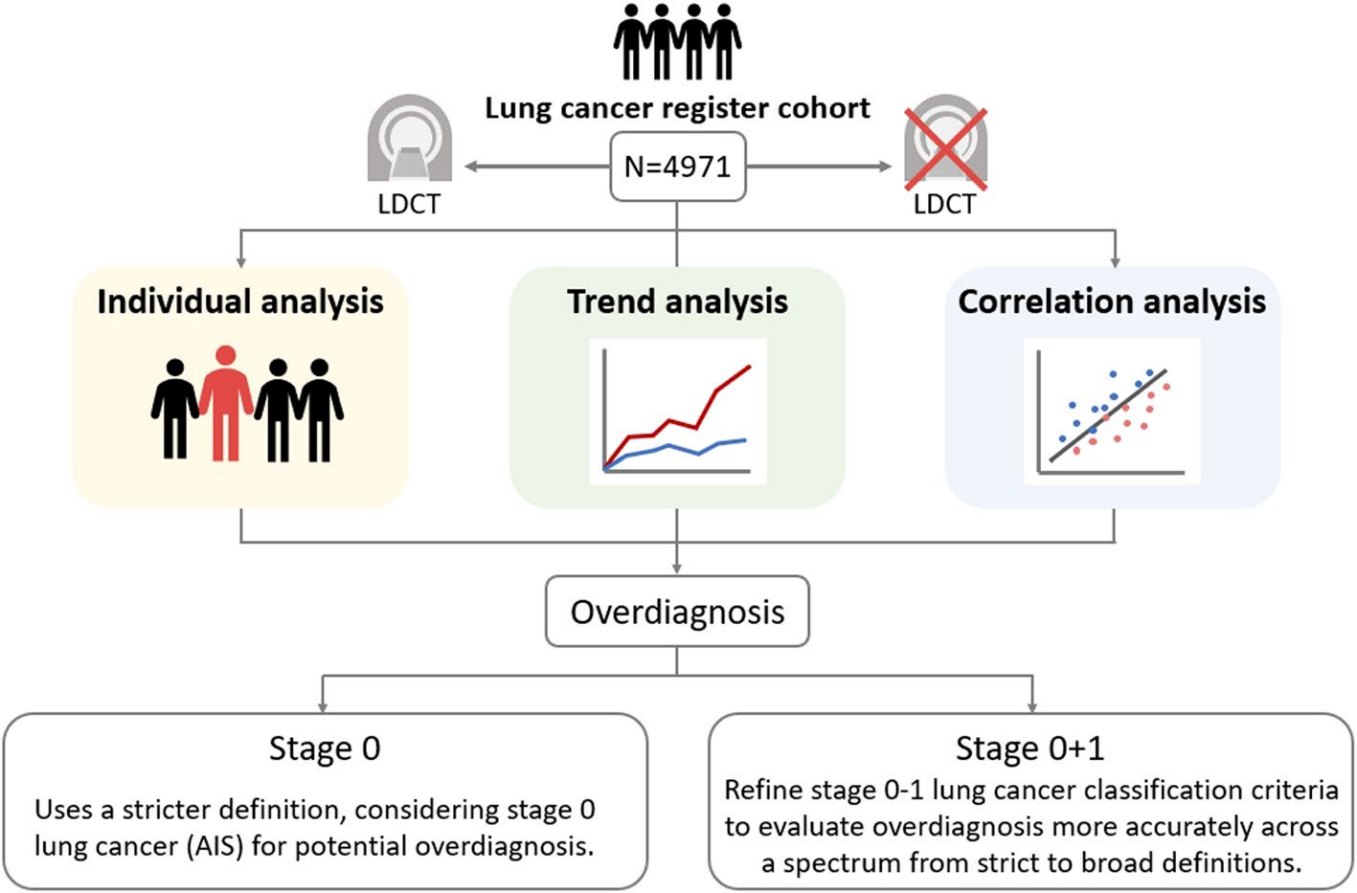
Flowchart depicting lung cancer register cohort study design to investigate trend analysis, individual and correlation analysis to address the issue of overdiagnosis

The study design was divided into three parts. In the first part, we employed a “year-trend based” approach to examine the trends in lung cancer screening volume and the characteristics of lung cancer screening over years. In the second part, using patients’ records, we analyzed the data of all patients with lung cancer registered in our hospital’s cancer registry database from 2008 to 2021 shown in Fig. 1. We linked these data with a list of all LDCT examinations in our hospital’s imaging database during the same period to investigate the impact of screening status on overdiagnosis. The third part involved visual chart analysis and Pearson correlation analysis to assess the trends in LDCT scan volume. We explored how these trends correlated with factors such as age and gender among patients with lung cancer, employing both visual and statistical analyses for evaluation.

### Definition of overdiagnosis

Overdiagnosis in healthcare involves diagnosing individuals with medical conditions or diseases that are not harmful or fatal, resulting in unnecessary medical interventions and overmanagement [12].

In real-world practice, managing lung nodules often requires shared decision-making between healthcare providers and patients, respecting patient preferences. Adhering to guidelines can be challenging, requiring clinicians to balance potential intervention benefits against the risks of overtreatment. In the real world, accurately determining whether early detection of lung cancer through screening results in overdiagnosis is challenging[8, 13]: the only way to confirm overdiagnosis in an individual is if that individual does not receive treatment and eventually does not develop lung cancer symptoms while succumbing to other causes. Most cases of early-stage lung cancer are treated or suspected of the patient having cancer; thus, assessing the exact rate of overdiagnosis is difficult. We cannot speculate whether individuals are overdiagnosed, but we can evaluate the potential overdiagnosis trend over time using lung cancer staging distribution through a hospital-based lung cancer register database and monitoring trends in the volume of LDCT scans [14–16]. In recent years, Asian countries such as South Korea, mainland China, and Taiwan have progressively discovered that, in terms of the definition of stage 0 to 1 lung cancers, applying LDCT lung cancer screening may lead to potential overdiagnosis, particularly among nonsmoking populations [8–10].

However, these studies utilized national lung cancer registry data and couldn't explore the correlation between real-world lung cancer screening implementation and potential overdiagnosis trends. Investigating if an individual's participation in screening is associated with potential overdiagnosis isn't feasible. This study linked a hospital-based lung cancer registry and imaging database, enabling further exploration of the correlation between screening status and potential overdiagnosis in a hospital-based cohort.

Localized disease or stage 0 to 1 lung cancer is used as a criterion for assessing potential overdiagnosis [8–10]. However, owing to the heterogeneity in the growth of lung adenocarcinoma, using these criteria may lead to overdiagnosis being overestimated. In the 8th International Association Study of Lung Cancer TNM classification staging project for lung cancer, individuals with adenocarcinoma in situ are categorized as having stage 0 disease, whereas those with minimally invasive adenocarcinoma are categorized as having stage IA1 [17]. At the individual patient-based level, we cannot directly determine whether minimally invasive adenocarcinoma or early lung cancers classified as stage IA2 and higher represent overdiagnosis. Therefore, this study adopted a more stringent definition of stage 0 lung cancer (adenocarcinoma in situ) as the standard for potential overdiagnosis. This study employed time-series trend analysis, correlation analysis, and individual-based logical analysis to investigate variations in the overdiagnosis of stage 0 early lung cancer associated with LDCT screening volumes across different time periods, genders, and age groups.

### Statistical analysis

Continuous data are reported as mean values accompanied by their respective standard deviations, whereas categorical data are reported as frequency distributions with their corresponding percentages. Statistical estimations were employed to visually represent the temporal trends. Descriptive statistical analyses were conducted using the lung cancer register database for each year from 2008 to 2021, with a particular focus on the clinical characteristics within the database. Utilizing data visualization methods, this study aimed to depict temporal trends in LDCT volume as part of an investigation into the potential overdiagnosis of stage 0 lung cancer. The categorization of data included factors such as gender, age range, and various time periods. In the current study, we explored the correlations between two quantitative variables with trends in LDCT volume and lung cancer register databases over time. Pearson correlations were employed for these analyses, with the significance level set at *P* < 0.05. All statistical analyses were performed using SPSS software version 18, developed by the IBM Corporation (Armonk, NY, USA). Logistic regression analysis was performed at the individual level to explore its association with stage 0 lung cancer. This study aimed to assess the possibility of overdiagnosis in the study cohort and. The analysis considered various clinical characteristics, including age, gender, lifestyle choices (such as smoking, and betel nut and alcohol consumption), and an individual’s screening status.

## Results

### Characteristics of lung cancer patients recorded in the cancer registry (2008 to 2021)

Table 1 displays yearly LDCT screening volumes and annual lung cancer statistics from 2008 to 2021, sourced from the hospital's cancer registry. It includes data on age, gender, lifestyle habits, stage distribution, behavior codes, lung cancer deaths, and survival rates at 1, 5, and 10 years, alongside the annual volume of LDCT exams conducted. The behavior code for lung cancer cases is assigned as malignant histology, whether in situ or invasive, in accordance with the pathology status [18]. Over this timeframe, 3,251 individuals within the hospital-based cohort in the specified target population were identified as having lung cancer. The lung cancer detection rate through LDCT screenings has gradually increased, rising from 1.3% in 2008 to 12.7% in 2021. Following the implementation of extensive LDCT examinations, the annual number of LDCT screenings in the hospital-based cohort exhibited a steady increase, starting at 245 in 2008 and reaching a peak of 3,079 in 2021. This signifies a remarkable 16-fold increase compared with the baseline period, with the most substantial growth observed between 2008 and 2019. The annual screening volume decreased from 2020 to 2021 due to isolation measures related to the coronavirus disease 2019 outbreak in Taiwan [19, 20].

**Table 1:** Characteristics of all patients diagnosed with lung cancer between 2008 and 2021 by sex, age, and period during gradual implementation of low-dose computed tomography screening in the hospital-based cohort

Year	2008	2009	2010	2011	2012	2013	2014	2015	2016	2017	2018	2019	2020	2021
Sex														
Male	222 (70.9%)	254 (69.6%)	201 (63.2%)	171 (60.6%)	200 (59.9%)	153 (54.1%)	199 (60.3%)	197 (55.6%)	188 (56.1%)	187 (53.3%)	200 (50.9%)	252 (57.1%)	186 (45.8%)	218 (46.8%)
Female	91 (29.1%)	111 (30.4%)	117 (36.8%)	111 (39.4%)	134 (40.1%)	130 (45.9%)	131 (39.7%)	157 (44.4%)	147 (43.9%)	164 (46.7%)	193 (49.1%)	189 (42.9%)	220 (54.2%)	248 (53.2%)
Age (yrs, mean ± SD)	69.05 ± 12.755	68.74 ± 12.562	67.71 ± 12.858	65.83 ± 13.747	66.12 ± 13.215	66.67 ± 12.099	66.91 ± 13.259	65.75 ± 12.387	63.80 ± 12.875	64.57 ± 11.834	62.61 ± 11.518	65.19 ± 10.385	64.62 ± 11.874	63.64 ± 11.481
Smoking	N/A	2 (50.0%)	1 (25.0%)	118 (42.4%)	149 (44.6%)	128 (45.6%)	164 (50.0%)	146 (41.5%)	136 (40.6%)	136 (39%)	141 (35.9%)	184 (41.8%)	142 (35.0%)	160 (34.3%)
Betel nut	N/A	1 (25.0%)	0	28 (10.1%)	32 (9.6%)	40 (14.3%)	45 (13.8%)	44 (12.5%)	41 (12.2%)	45 (12.9%)	52 (13.2%)	56 (12.7%)	45 (11.1%)	55 (11.8%)
Alcohol drinking	N/A	2 (50%)	0	64 (23.2%)	96 (28.8%)	89 (31.9%)	98 (30.0%)	93 (26.7%)	75 (22.8%)	94 (27.2%)	91 (23.3%)	110 (25.2%)	97 (24.1%)	115 (24.9%)
Stage distribution														
Stage 0	0	1(0.3%)	0	0	1 (0.3%)	1 (0.4%)	6 (1.8%)	4 (1.1%)	12 (3.6%)	17 (4.9%)	26 (6.6%)	24 (5.5%)	19 (4.7%)	50 (10.7%)
Stage I	35 (11.6%)	65 (17.9%)	42 (13.3%)	36 (12.9%)	65 (19.5%)	63 (22.3%)	74 (22.5%)	98 (27.7%)	85 (25.4%)	100 (28.6%)	121 (30.8%)	158 (36.0%)	153 (37.7%)	188 (40.3%)
Stage II	9 (3.0%)	12 (3.3%)	13 (4.1%)	17 (6.1%)	15 (4.5%)	14 (4.9%)	18 (5.5%)	15 (4.2%)	16 (4.8%)	9 (2.6%)	24 (6.1%)	26 (5.9%)	14 (3.4%)	6 (1.3%)
Stage III	120 (39.6%)	108 (29.8%)	67 (21.2%)	51 (18.3%)	52 (15.6%)	42 (14.8%)	51 (15.5%)	44 (12.4%)	47 (14.0%)	36 (10.3%)	51 (13.0%)	45 (10.3%)	46 (11.3%)	35 (7.5%)
Stage IV	139 (45.9%)	177 (48.8%)	194 (61.4%)	175 (62.7%)	201 (60.2%)	163 (57.6%)	180 (54.7%)	193 (54.5%)	175 (52.2%)	188 (53.7%)	171 (43.5%)	186 (42.4%)	174 (42.9%)	187 (40.1%)
Behavior code														
Carcinoma in situ		1 (0.3%)			1 (0.3%)	1 (0.4%)	6 (1.8%)	4 (1.1%)	12 (3.6%)	17 (4.8%)	26 (6.6%)	24 (5.4%)	19 (4.7%)	50 (10.7%)
Invasive cancer	313 (100%)	364 (99.7%)	318 (100%)	282 (100%)	333 (99.7%)	282 (99.6%)	324 (98.2%)	350 (98.9%)	323 (96.4%)	334 (95.2%)	367 (93.4%)	417 (94.6%)	387 (95.3%)	416 (89.3%)
Lung cancer number	313	365	318	282	334	283	330	354	335	351	393	441	406	466
Lung cancer number (screened^a^)	4 (1.3%)	1 (0.3%)	0	3 (1.1%)	3 (0.9%)	12 (4.2%)	13 (3.9%)	35 (9.9%)	26 (7.8%)	30 (8.5%)	39 (9.9%)	42 (9.5%)	45 (11.1%)	59 (12.7%)
Lung cancer death	292 (93.3%)	338 (92.6%)	297 (93.4%)	249 (88.3%)	283 (84.7%)	222 (78.4%)	253 (76.7%)	248 (70.1%)	225 (67.2%)	217 (61.8%)	202 (51.4%)	203 (46.0%)	158 (38.9%)	120 (25.8%)
1-year survival rate	53.74%	50.68%	55.08%	62.41%	62.87%	63.59%	66.97%	65.18%	70.61%	74.78%	77.13%	75.18%	76.48%	79.92%
95% CI	0.4804–0.5909	0.4545–0.5568	0.4943–0.6037	0.5648–0.6777	0.5745–0.6781	0.5769–0.6890	0.6161–0.7176	0.5996–0.6990	0.6540–0.7519	0.6988–0.7901	0.7262–0.8099	0.7086–0.7895	0.7200–0.8034	0.7589–0.8335
5-year survival rate	16.41%	18.87%	15.17%	19.27%	25.34%	25.74%	30.27%	31.99%	34.38%	38.23%	45.90%	-	-	-
95% CI	0.1254–0.2074	0.1504–0.2304	0.1147–0.1936	0.1489–0.2410	0.2079–0.3013	0.2078–0.3097	0.2539–0.3528	0.2716–0.3691	0.2928–0.3952	0.3307–0.4337	0.4042–0.5120	-	-	-
10-year survival rate	9.50%	8.37%	7.73%	12.03%	15.26%	20.19%	-	-	-	-	-	-	-	-
95% CI	0.0655–0.1309	0.0572–0.1164	0.0504–0.1115	0.0849–0.1622	0.1159–0.1940	0.1554–0.2528	-	-	-	-	-	-	-	-
LDCT number	245 (0.86%)	434 (1.52%)	352 (1.23%)	596 (2.09%)	1006 (3.53%)	1119 (3.92%)	1501 (5.26%)	2810 (9.85%)	2879 (10.09%)	3236 (11.34%)	3723 (13.05%)	4005 (14.04%)	3543 (12.42%)	3079 (10.79%)

*Abbreviations*: *SD* standard deviation, *CI* confidence interval, *LDCT* low-dose computed tomography

^a^Yearly rate of screen-detected lung cancers

**Fig. 2:**
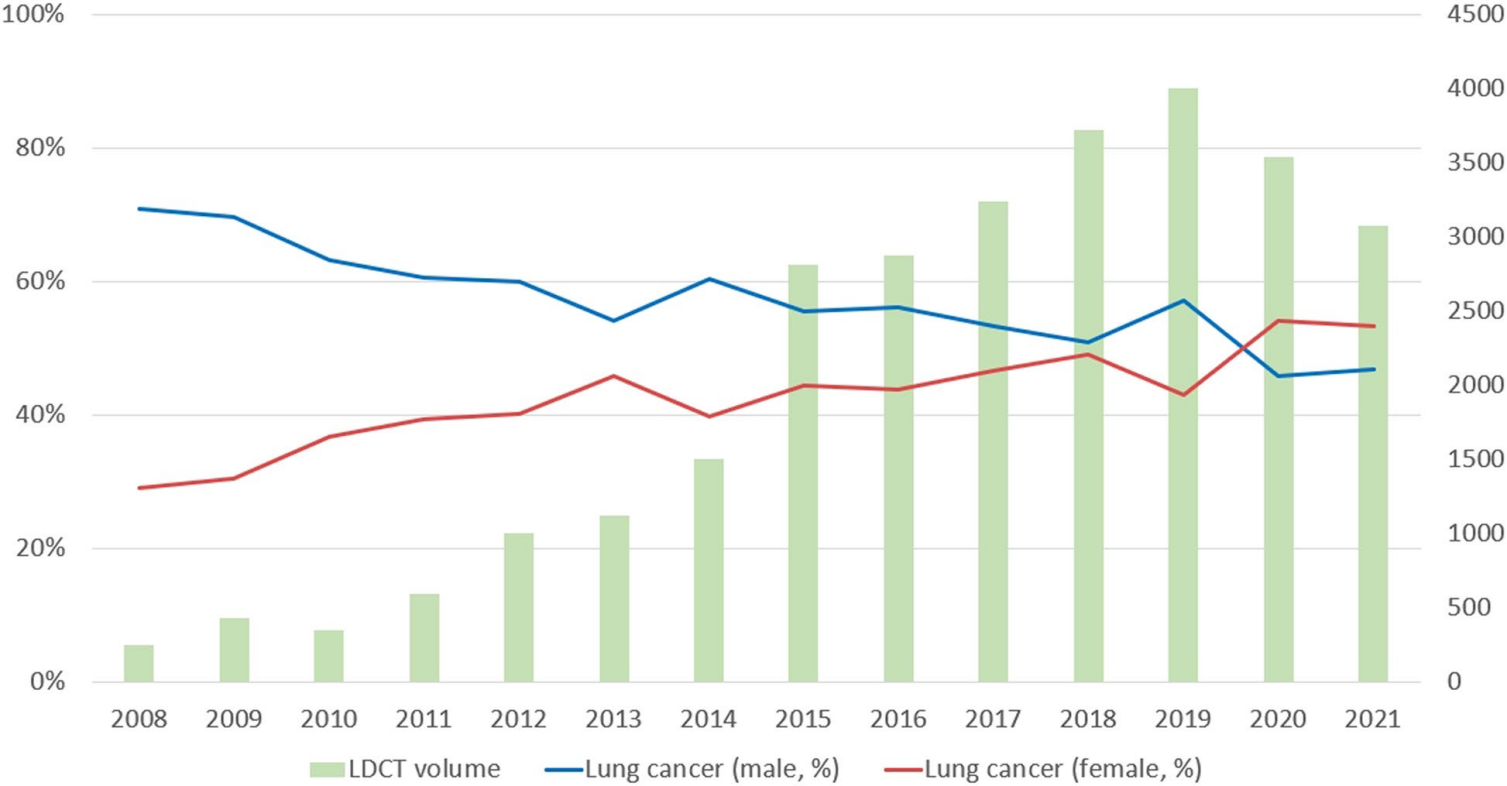
Trends in LDCT examination volumes and percentage of Lung Cancer by Gender from 2008 to 2021

**Fig. 3:**
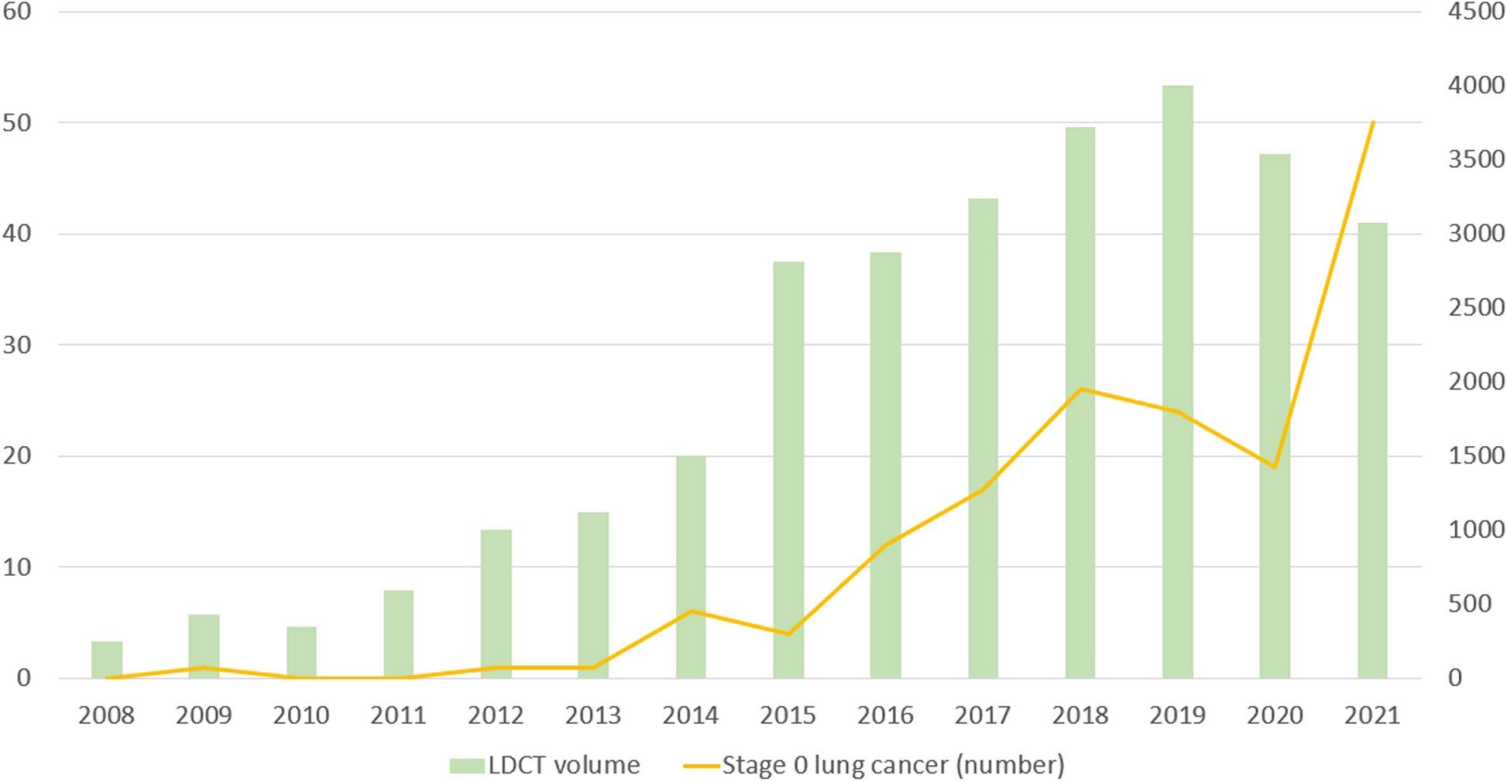
Trends in LDCT examination volumes and number of stage 0 Lung Cancer from 2008 to 2021

Between 2008 and 2021, the proportion of male lung cancer patients declined gradually from 70.9% to 46.8%, while the percentage of female patients increased from 29.1% to 53.2% (Fig. 2). The average age of lung cancer diagnosis decreased from 69.05 ± 12.755 years in 2008 to 63.64 ± 11.481 years in 2021. Figure 3 displays a trend analysis of LDCT screening volumes and stage 0 lung cancer cases from 2008 to 2021: from 2008 to 2019, both increased, reflecting a positive correlation. A decline occurred in 2020 due to the COVID-19 outbreak, followed by a significant rise in 2021 as pandemic conditions eased, further highlighting the correlation. The data also show a consistent increase in the percentage of stage 0 cases from 2008 to 2019, mirroring the rise in LDCT screenings.

**Fig. 4:**
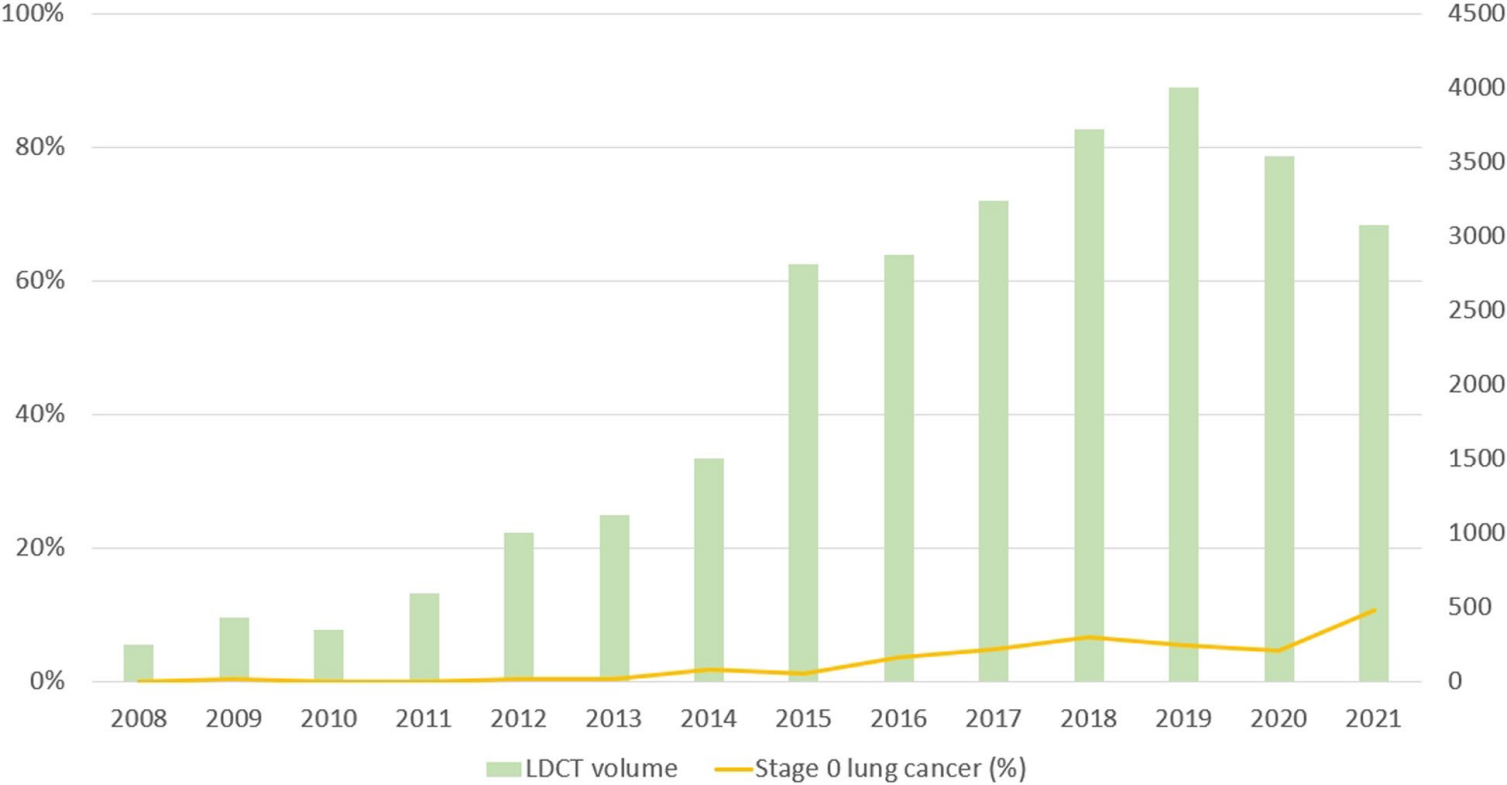
Trends in LDCT examination volumes and percentage of stage 0 Lung Cancer from 2008 to 2021

However, from 2019 to 2020, owing to the impact of the coronavirus disease 2019 pandemic, both the LDCT screening volume and the percentage of stage 0 lung cancer cases decreased slightly. Only with the easing of the pandemic in 2020 to 2021, did both indicators show a gradual upward trend (Fig. 4). The number of stage 0 lung cancers was minimal during the period from 2008 to 2013, with only sporadic three cases detected due to limited screening. However, starting from 2014, with the increasing volume of screenings over the years, the count of stage 0 lung cancers rose from six in 2014 to fifty in 2021.

### Correlation between lung cancer registry trend and LDCT scan volume

In this hospital cohort study, we explored the relationship between evolving lung cancer characteristics and LDCT volumes. Notably, we observed negative correlations between the annual changes regarding age at lung cancer diagnosis and LDCT screening volume (*r* =  − 0.843, *P* < 0.001) and between the annual trend in lung cancer death rates and LDCT implementation (*r* =  − 0.778, *P* = 0.001). Furthermore, negative correlations were observed between the number and percentage of stage 3 lung cancer cases and annual LDCT screening volume (*r* =  − 0.637, *P* = 0.014 and *r* =  − 0.753, *P* = 0.002, respectively) shown in Table 2.

**Table 2:** Investigating the relationship between changes in lung cancer characteristics over time and the introduction of low-dose computed tomography in a hospital cohort

	LDCT exam volume
Age (yrs, mean)	-0.843 (< 0.001)
Lung cancer number	0.730 (0.003)
1-year survival rate	0.912 (< 0.001)
5-year survival rate	0.964 (< 0.001)
10-year survival rate	0.939 (0.005)
Lung cancer death	-0.778 (0.001)
Stage 0 (number)	0.747 (0.002)
Stage I (number)	0.861 (< 0.001)
Stage II (number)	0.352 (0.217)
Stage III (number)	-0.637 (0.014)
Stage IV (number)	0.235 (0.418)
Stage 0 (%)	0.801 (0.001)
Stage I (%)	0.912 (< 0.001)
Stage II (%)	0.011 (0.971)
Stage III (%)	-0.753 (0.002)
Stage IV (%)	-0.615 (0.019)

*Abbreviations*: *LDCT* low-dose computed tomography

In summary, our analysis revealed several significant correlations. We found a positive association between the annual increase in diagnosed lung cancer cases and LDCT screening volume (*r* = 0.730, *P* = 0.003). Similarly, a positive relationship was observed between LDCT screening volume and 1, 5, and 10-year survival rates (*r* = 0.912, *P* < 0.001; *r* = 0.964, *P* < 0.001; and *r* = 0.939, *P* = 0.005; respectively). Furthermore, positive associations were noted between the number and proportion of stage 0 lung cancer cases and annual LDCT screenings volume (*r* = 0.747, *P* = 0.002 and *r* = 0.801, *P* = 0.001, respectively). Positive correlations were observed between the number and proportion of stage 1 cases and annual LDCT screenings volume (*r* = 0.861, *P* < 0.001 and *r* = 0.912, *P* < 0.001, respectively).

### Data visualization for differential trend periods and gender effect

**Fig. 5:**
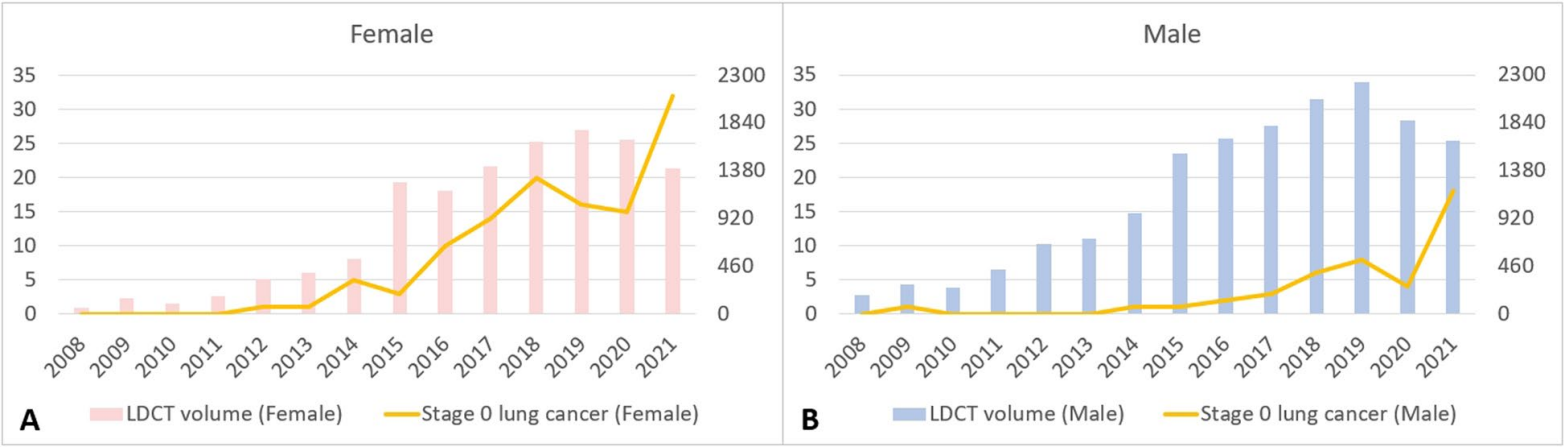
Trends in LDCT examination volumes and number of stage 0 Lung Cancer by gender from 2008 to 2021

Figure 5 compares LDCT screening volumes and stage 0 lung cancer cases from 2008 to 2021 by gender. Males consistently underwent more LDCT screenings, but females consistently had more stage 0 diagnoses; both charts share the same y-axis scale. This highlights LDCT screening's greater impact in increasing stage 0 lung cancer cases in females compared to males.

**Fig. 6:**
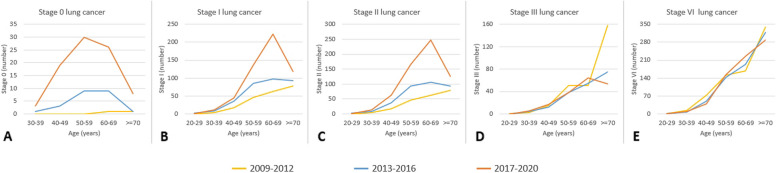
Exploring the changing distribution of lung cancer cases (stage 0 to stage 4) across different age groups in three distinct time periods (2009–2012, 2013–2016, 2017–2020)

Figure 6 shows the fluctuating trends in the impact of LDCT examination volumes on various stages of lung cancer over the different time periods, highlighting the corresponding changes in diverse age groups. For stage 0 lung cancer, the table reveals a progressive increase in the LDCT scan volume over time. Notably, from 2017 to 2020, a significantly more pronounced increase in the number of stage 0 lung cancer cases across various age groups was noted compared with the periods 2013 to 2016 and 2009 to 2012. Additionally, the grouping by age exhibited a normal distribution pattern centered around an average age of 50 years. Regarding the variation in stage 1 lung cancer cases, LDCT scan volumes increased over time. Specifically, from 2017 to 2020, the rise in stage 1 cases across age groups was notably more significant compared to 2013 to 2016 and 2009 to 2012. The largest increase occurred in the 50 to 70 age range, with a slower rate of increase in the 30 to 50 age range. Examining the changes in the number of stage 2 lung cancer cases revealed that, as the LDCT scan volume increased over time, so the number of stage 2 lung cancer cases among individuals aged 50 to 70 years gradually increased from 2017 to 2020. Conversely, within the age range of 20 to 50 years, the data indicated relatively stable numbers of stage 2 lung cancer cases over this 3-year period. Analyzing changes in stage 3 lung cancer cases showed a decline among individuals aged 60 to 70 as LDCT scan volume increased from 2017 to 2020. Conversely, within 20 to 60 years, numbers remained relatively stable over the 3-year period. Exploring stage 4 cases, data indicated stability across age groups over the three periods.

**Fig. 7:**
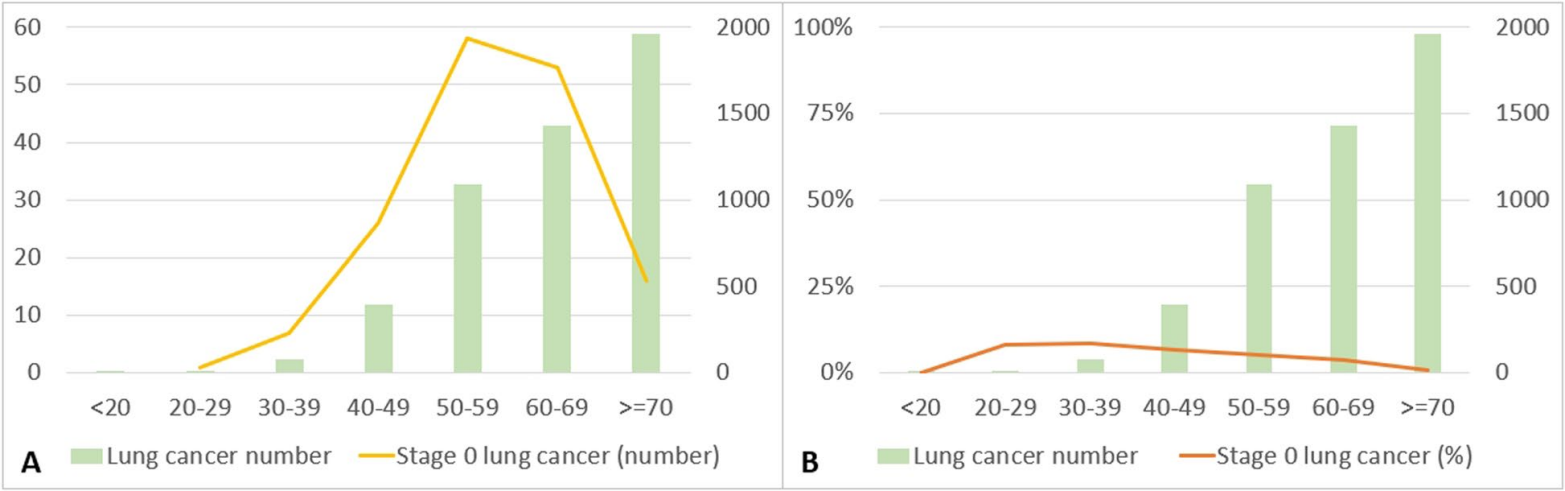
Changes in the number and percentage of stage 0 lung cancer in different age groups from 2008 to 2021

When investigating the changes in the number of stage 0 lung cancer cases across different age groups from 2008 to 2021, stage 0 lung cancer cases were distinctly most prevalent in the age range of 40 to 70 years. However, when looking at the distribution of stage 0 lung cancer cases as a percentage of all diagnosed lung cancer cases, the highest proportion was observed in the 20 to 40-year age group (Fig. 7).

**Fig. 8:**
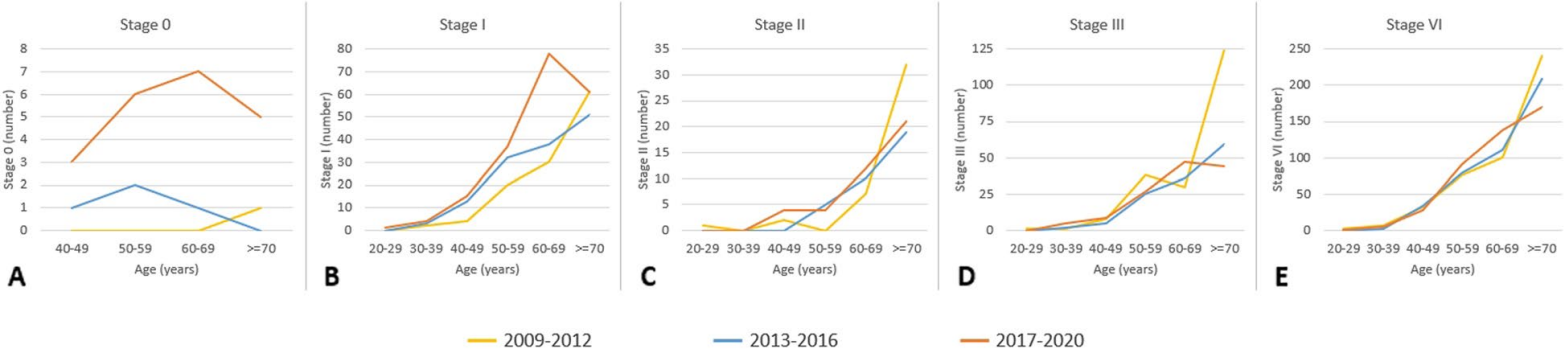
Exploring the changing distribution of lung cancer cases (stage 0 to stage 4) across different age groups among men in three distinct time periods (2009–2012, 2013–2016, 2017–2020)

**Fig. 9:**
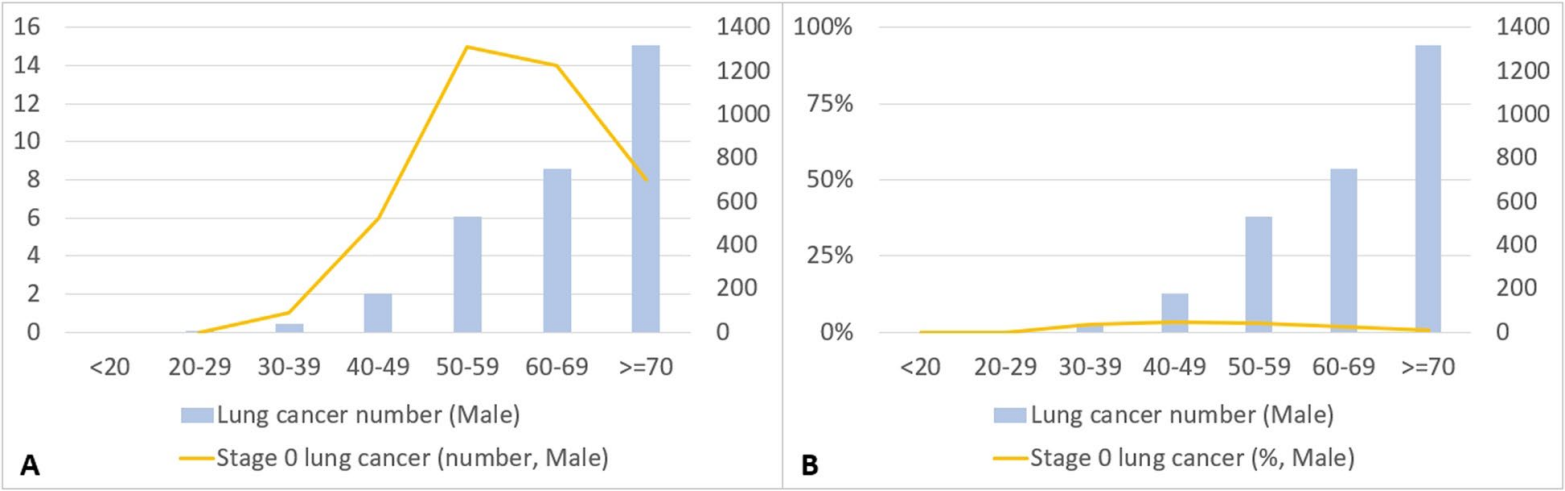
Changes in the number and percentage of stage 0 lung cancer among men in different age groups from 2008 to 2021

Figures 8 and 9 show the fluctuating trends in the impact of LDCT examination volumes on various stages of lung cancer over different time periods, highlighting the corresponding changes in diverse age groups among males. Figure 8 shows the variations in the number of stage 0 lung cancers among males of different ages during the three distinct periods from 2009 to 2020. The volume of LDCT scans clearly increased over time. Stage 0 lung cancers across different age groups exhibited a significantly greater increase in numbers in the period 2017 to 2020 compared with the periods 2013 to 2016 and 2009 to 2012. The age distribution showed a normal increase.

Next, we examined changes in the number of stage 1 lung cancers among males. A significantly greater increase in the number of stage 1 lung cancers among the different age groups was evident in the period 2017 to 2020 than in the two periods from 2009 to 2016. The group of males aged 50 to 70 years showed the highest increase, whereas the group aged 30 to 50 years displayed a relatively slower rate of increase. In the investigation of stage 2 lung cancers among males, a clear increase over time was noted in the volume of LDCT scans. From 2017 to 2020, the number of stage 2 lung cancers increased gradually among those aged 50 to 70 years. However, among those older than 70 years, a slight decrease in the number of stage 2 lung cancers was observed over this 3-year period.

The exploration of changes in the number of stage 3 lung cancers among males showed a clear increase in the volume of LDCT scans over time. In the period 2017 to 2020, the number of stage 3 lung cancers showed a declining trend among males aged between 60 and 70 years. However, in the 20 to 60-year age group, the number of stage 3 lung cancers remained relatively stable over the 3-year period. Finally, we investigated the changes in the number of stage 4 lung cancers among males: the volume of LDCT scans clearly increased over time. The data showed that from 2017 to 2020, the number of stage 4 lung cancers among males aged 50 to 70 years gradually increased. Conversely, in those older than 70 years, a slight decrease in the number of stage 4 lung cancers was observed during the same period. The number of stage 4 lung cancers among males aged between 20 and 50 years remained relatively stable over the three different years.

We investigated changes in the number of stage 0 lung cancers among males of different age groups from 2008 to 2021. Stage 0 lung cancer was most prevalent in males aged 40 to 70 years. When examining the distribution of stage 0 lung cancer cases as a percentage, the group aged between 40 and 49 years had the highest proportion of stage 0 lung cancer cases among the overall number of diagnosed lung cancer cases (Fig. 9).

**Fig. 10:**
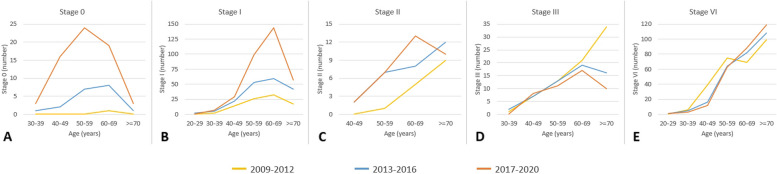
Exploring the changing distribution of lung cancer cases (stage 0 to stage 4) across different age groups among women in three distinct time periods (2009–2012, 2013–2016, 2017–2020)

**Fig. 11:**
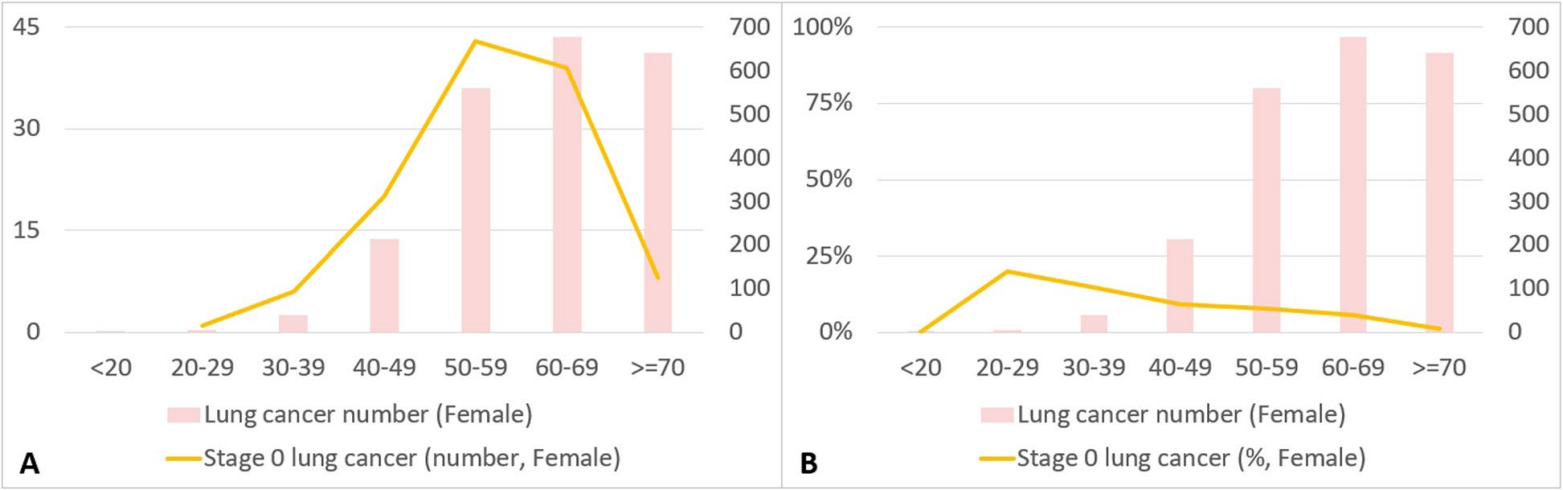
Changes in the number and percentage of stage 0 lung cancer among women in different age groups from 2008 to 2021

The fluctuating trends in the impact of LDCT examination volumes on various stages of lung cancer over different time periods are presented in Figs. 10 and 11, highlighting the corresponding changes in different age groups among females. Figure 10 shows the results of our investigation of the changes in the number of stage 0 lung cancers among females of different age groups during the three periods from 2008 to 2021. The volume of LDCT scans clearly increased over time. From 2017 to 2020, a significantly greater increase in the number of stage 0 lung cancers across different age groups was observed compared with the periods of 2013 to 2016 and 2009 to 2012. The age distribution was normal. Next, we examined changes in the number of stage 1 lung cancers among females. The volume of LDCT scans clearly increased over time: from 2017 to 2020, a significantly greater increase in the number of stage 1 lung cancers among different age groups was observed compared with the other two periods The age distribution showed that the highest increase occurred among females aged between 50 and 70 years, whereas a relatively slower rate of increase occurred among females aged between 30 and 50 years. In our investigation of stage 2 lung cancers among females, compared with the period from 2009 to 2016, a notable increase was evident in the number of stage 2 lung cancers among those aged between 60 and 69 years in the period from 2017 to 2020. However, considering the various age groups from 40 to 70 years, the number of stage 2 lung cancers increased across all age groups as time progressed. Our investigation of changes in stage 3 lung cancers among females showed a decline in cases among those over 60. However, among females aged 30 to 69, numbers remained relatively stable over 3 years. Investigating stage 4 lung cancers among females, data revealed a decrease in cases among those aged 30 to 49 from 2013 to 2020 compared to 2009 to 2012. However, from 2013 to 2020, changes in stage 4 lung cancers across age groups were relatively stable.

Figure 11 shows the changes in the number of stage 0 lung cancers among females of different age groups from 2008 to 2021. Stage 0 lung cancer was most prevalent in females aged between 40 and 70 years. When examining the distribution of stage 0 lung cancer cases as a percentage of the overall diagnosed lung cancer cases, a significantly higher proportion of stage 0 lung cancer cases was evident among females aged between 20 and 39 years than among those in the other age groups.

### Estimated overdiagnosis

We utilized logistic regression to investigate the correlation between individual-level clinical risk factors and stage 0 lung cancer to analyze their impact on potential overdiagnosis. Multivariate logistic regression analysis showed that age, smoking status, and screening status remained significantly associated with stage 0 lung cancer after adjusting for confounding factors (Table 3). Screened status was a strong independent risk factor for stage 0 lung cancer, with an adjusted odds ratio of 7.617 (95% confidence interval, 5.357–10.831; *P* < 0.001), suggesting a potential overdiagnosis.

**Table 3:** Logistic regression analysis of clinical parameters for the prediction of patients with stage 0 lung cancer

Variable	Coefficient	OR	95% CI	*P*-value
Age (years)	-0.048	0.953	0.939–0.967	< 0.001
Sex (reference: male)	0.342	1.408	0.899–2.203	0.135
Smoking	-0.851	0.427	0.228–0.799	0.008
Betel nut	-0.55	0.577	0.190–1.753	0.332
Alcohol drinking	-0.215	0.806	0.447–1.456	0.476
Screened status^a^	2.03	7.617	5.357–10.831	< 0.001

*Abbreviations*: *OR* Odd Ratio, *CI* Confidence interval

^a^Screened status: The control group includes those who have not received lung cancer screening, and the experimental group includes those who have received lung cancer screening

To further assess the degree of overdiagnosis, we employed a definition of stages 0 to 1 lung cancer. Adoption of this stage 0 to 1 lung cancer definition was based on previous literature reviews and can serve as a broad standard for defining overdiagnosis. However, as we cannot definitively ascertain that all first-stage lung cancers are cases of overdiagnosis at the individual level, using this definition may lead to an overestimation of the extent of overdiagnosis. Furthermore, in the logical analysis, we utilized lung cancer stages 0 to 1 as the outcome events. The multivariate logistic regression analysis indicated that factors such as age, smoking status, female gender, and screening status remained significantly correlated with stage 0 to 1 lung cancer, even after adjusting for confounding factors. Notably, screening status emerged as a robust independent risk factor for stage 0 to 1 lung cancer, with an adjusted odds ratio of 17.114 (95% confidence interval, 11.752–25.011; *P* < 0.001) shown in Table 4. These findings suggest a more substantial potential for overdiagnosis in the context of stage 0 to 1 lung cancer compared with the context of a definition based solely on stage 0.

**Table 4:** Logistic regression analysis of clinical parameters for the prediction of patients with stage 0 + 1 lung cancer

Variable	Coefficient	OR	95% CI	*P*-value
Age (years)	-0.025	0.975	0.969–0.981	< 0.001
Sex (reference: male)	0.421	1.523	1.255–1.849	< 0.001
Smoking	-0.840	0.432	0.344–0.542	< 0.001
Betel nut	-0.291	0.747	0.554–1.008	0.057
Alcohol drinking	0.174	1.190	0.945–1.500	0.139
Screened status^a^	2.842	17.144	11.752–25.011	< 0.001

*Abbreviations*: *OR* Odd Ratio, *CI* Confidence interval

^a^Screened status: The control group includes those who have not received lung cancer screening, and the experimental group includes those who have received lung cancer screening

## Discussion

This study used a hospital-based cohort to investigate the impact of long-term LDCT screening volumes on a lung cancer register database. We observed the long-term implementation of LDCT screening can lead to a shift in lung cancer staging to early-stage lung cancer and a year-on-year increase in overall lung cancer survival rates. This study investigated the real-world impact in nonsmoking and smoking individuals of Asian descent, considering factors such as anxiety and the lack of targeted screening due to the challenges of defining a high-risk Asian population [8]. This screening is undertaken on a largely voluntary basis and at the individual’s own expense, often prompted by concerns related to a personal or family history of lung cancer and the associated anxieties [21].

In recent years, the extensive use of LDCT in nonsmoking Asian populations has given rise to a growing issue of overdiagnosis in real-world scenarios, which is evident in countries such as mainland China, South Korea, and Taiwan [8–10]. However, research examining how the prolonged implementation of LDCT volume affects a lung cancer register database cohort and its impact on overdiagnosis is lacking. To the best of our knowledge, the present study is the first retrospective investigation of the long-term effects of implementing a high volume of LDCT on the detection of stage 0 lung cancer to address the issue of overdiagnosis in the Asian population. This study had three key findings. First, with the increasing volume of lung cancer screening over the years, the number and proportion of stage 0 lung cancers have grown proportionally. This demonstrates a significant relationship between the number of LDCT scans and risk of overdiagnosis. Second, we explored the relationship between the LDCT scan volume and different time periods and different stages of lung cancer. The results indicated that stage 0 lung cancer increased with the growth trend in lung cancer screening volumes, particularly during the period from 2017 to 2020, in which the most significant growth took place. In contrast, the number of stage 4 lung cancer cases remained stable through the different time intervals, suggesting that the extensive use of LDCT significantly increases the risk of overdiagnosis of stage 0 lung cancer. Third, our study examined the impact of gender differences on the relationship between lung cancer screening volume and overdiagnosis. The results of this analysis revealed that the growth in lung cancer screening volume had a significantly higher impact on the increase in the number of stage 0 lung cancer cases in females than it had in males. This indicates that the increasing implementation of LDCT is likely to result in a noticeable overdiagnosis of stage 0 lung cancer in females. Fourth, the results of the logistic statistical analysis indicated an association between the presence of stage 0 lung cancer and younger age, nonsmoking status, participation with screened status ( +), and potential overdiagnosis. The current study successfully addressed the knowledge gap concerning how the volume of LDCT examinations affects overdiagnosis, particularly in the context of stage 0 lung cancer detection. Across the spectrum of different definitions encompassing both stage 0 and stage 0 to 1 lung cancer, undergoing screening, as opposed to not undergoing screening, markedly increases the probability of potential overdiagnosis. This was evidenced by odds ratios of 7.5 and 17 for undergoing screening and not undergoing screening, respectively, indicating varying degrees of impact. This study highlights a robust effect, with an odds ratio ranging from 7 to 17 indicating that LDCT screening status significantly affects the degree of potential overdiagnosis, even under a more stringent definition of overdiagnosis (stage 0 lung cancer).

Previously, stage 1 or localized stage lung cancer were used to assess the extent of overdiagnosis [8–10, 22, 23]. Using such a definition tends to overestimate the extent of overdiagnosis as, from an individual lung cancer perspective, we cannot determine whether early-stage lung cancer treated with surgery is indeed over diagnosed. This study adopted the more rigorous definition of stage 0 lung cancer to determine the extent of overdiagnosis. The annual trend analysis results indicated a positive correlation between the volume of LDCT scans and the incidence of stage 0 lung cancer, with a significantly greater impact on females than on males. Females younger than 40 years old were especially impacted. The best way to control overdiagnosis is to stop performing lung cancer screenings altogether [24]. However, this is impractical in Asian countries that have a high prevalence of nonsmoking lung cancer. Many individuals voluntarily participate in self-funded lung cancer screenings because of the anxiety caused by their family members’ nonsmoking lung cancer-related deaths [8]. While overdiagnosis is an inevitable outcome of implementing screening programs, the present study found that, with an increase in the volume of screening over time, the degree of overdiagnosis has become more pronounced. In Asian countries with a high prevalence of nonsmoking-related lung cancer, the root causes of overdiagnosis resulting from lung cancer screening are inherently complex [8–10]. Based on the findings of this real-world study, we intended to analyze the fundamental reasons at the policy, healthcare provider, and patient levels and to develop policy, healthcare-professional, and individual-level strategies to improve the incidence of overdiagnosis by addressing its underlying causes.

From a government policy perspective, utilizing a lung cancer risk prediction model to establish screening thresholds can optimize screening efficiency [21, 25–29]. Targeting individuals with higher percentile levels of lung cancer risk for screening increases the probability of obtaining benefits from screening [29, 30]. Therefore, using a lung cancer risk prediction model to define the screening population may be more effective in enhancing the benefits of lung cancer screening than the binary criteria standards commonly used at present [31]. To reduce the potential for overdiagnosis, especially among women younger than 40 years, implementing more rigorous risk prediction models and considering raising the screening age threshold for nonsmoking female populations when it comes to lung cancer screening is advised [21]. Heavy smokers may be less willing to participate in lung cancer screening programs [32]; therefore, increasing screening rates among heavy smoking populations is an important issue [21]. However, for nonsmoking populations in Asia, where concerns about lung cancer are collective, screening efficiency must be optimized based on risk-prediction models tailored for nonsmokers and health education must be provided to reduce screening reluctance and anxiety among this group [21].

From the perspective of healthcare service providers, continuing education programs for professionals such as general practitioners, pulmonologists, thoracic surgeons, and radiologists are essential to help them understand that overdiagnosis often results from overtreatment [33]. Therefore, educating healthcare professionals on adopting an active surveillance approach for ground-glass nodules and defining higher growth thresholds (increasing the threshold from 0.2 cm to 0.5 cm) in the monitoring process to reduce the rate of excessive use of surgical interventions for stage 0 lung cancer is crucial [33, 34]. Additionally, a group of physicians is concerned that these slow-growing lung cancers or precancerous lesions may cause patients to seek multiple opinions at different hospitals and subsequently receive a lung cancer diagnosis elsewhere. This practice can damage the relationship of trust between patient and doctor and potentially result in medical malpractice lawsuits. This is because advising patients to undergo regular active monitoring only for those diagnosed with lung cancer and treated surgically at another hospital often raises retrospective questions in the physician’s career and leads to unnecessary conflict. Surgeons must understand that overdiagnosis results from excessive intervention in the real world. While we are aware that recent advances in thoracic surgery and minimally invasive techniques have significantly improved the safety of early-stage lung cancer surgeries and reduced complications, adopting an active monitoring strategy until the patient meets the surgical criteria is the best approach to reduce overdiagnosis [35, 36].

Radiologists should aim to avoid false positives or ineffective high Lung-RADS grading reports and consider using instead higher growth threshold values to reduce the likelihood of surgical intervention for ground-glass nodules [37].

At the individual level, the public’s understanding of the potential benefits and drawbacks of lung cancer screening, primarily based on their personal lung cancer risk, is important [38]. Through health education and shared decision-making, people can improve their understanding and knowledge of lung cancer screening [33, 39]. This can help reduce anxiety among individuals who are faced with indeterminate lung nodules and encourage them to engage in long-term active surveillance until they reach the clinical threshold or indications for surgery recommended by healthcare professionals.

The key strength of the present study lies in its access to a meticulously maintained cancer registry in a hospital setting that provides detailed information on individuals diagnosed with lung cancer. These data were complemented by a dataset that tracked the annual trends in LDCT examination volumes from an image database. Notably, the study followed a cohort of patients for up to 14 years, allowing for a thorough analysis of how overdiagnosis rates have evolved over time and how the distribution of cancer stages has shifted following the introduction of the lung cancer screening program. In essence, this research represents a long-term time-series analysis that seeks to uncover the relationship between the volume of LDCT examinations and the potential for the overdiagnosis of stage 0 lung cancer. Its objective was to delve into the causality behind this phenomenon and assess whether the extension of the screening period has contributed to a growing tendency toward overdiagnosis.

This study had several limitations. First, some clinical information, such as smoking habits and betel and alcohol consumption status, was incomplete, resulting in bias. Some information could not be extracted from the hospital-based cancer registry. We acknowledge that some screening participants may seek healthcare at facilities outside our hospital, potentially resulting in gaps in our data. Additionally, we recognize the growing popularity of sublobar lung surgery as a less invasive option and the increasing emphasis on shared decision-making in treatment choices. These factors could significantly impact the management strategies employed and, consequently, the outcomes of our study Additionally, we recognize the growing popularity of sublobar lung surgery as a less invasive option and the increasing emphasis on shared decision-making in treatment choices. Second, the screening coverage was insufficient due to the lack of a national lung cancer screening program in Taiwan during the study period. In the current study, we observed that the number of stage 1 lung cancer cases has increased annually. The proportion of stage 4 cases has decreased, but not significantly decreased in the number of stage 4 lung cancer. The trend we observed could potentially be explained by insufficient screening coverage and the likelihood of overdiagnosis in the epidemic area. This study observed an increase in the number of early-stage cases over several years of screening, indicating the annual increase in screening volume has led to a phenomenon known as “stage shift.”[40] This, in turn, further improved the overall lung cancer mortality rate. However, according to the literature, “stage shift” is not a reliable predictor of mortality benefit [41]. Evaluating the reduction in the number of advanced stage (stage 4) lung cancer cases is also a crucial factor in assessing the mortality benefits of screening [42].

Third, the present study may not be generalizable to other hospitals or countries in Asia given the predominant focus on self-paid lung cancer screening in the study’s design. However, this study more accurately depicted the worsening phenomenon of overdiagnosis resulting from the increased uptake of lung cancer screening due to collective anxiety regarding lung cancer in the real world. Thus, further large randomized controlled studies are needed to investigate the effect of implementing LDCT screening programs in target risk populations among nonsmokers in Asia. Forth, we understand our study differs from the NLST and NELSON cohorts, particularly regarding smoking history and age criteria. Our focus on a real-world Asian population aged 40 to 80 years, including self-paid participants, distinguishes our study from the NLST's emphasis on a smoking population. These distinctions introduce unique limitations to our research. To address this, we aim for a nuanced analysis that considers these intricacies for a more accurate data interpretation. Rather than using standardized lung cancer incidence rates, we chose to analyze data from a single hospital cohort over time. This decision may limit our study by not providing standardized incidence rates for comparison and not accounting for environmental factors that may contribute to the rise in lung cancer cases. Fifth, it's important to note that our focus on invasive lung cancer among female non-smokers in Asia may come with inherent challenges. Our understanding of its natural history remains limited, and there's a possibility that this could represent a novel form of lung cancer. Therefore, a longer follow-up period may be necessary to achieve a more comprehensive epidemiological understanding.

## Conclusion

These findings suggest the gradual implementation of an LDCT lung screening program in this hospital-based cohort may have led to a significant increase in the potential overdiagnosis of stage 0 lung cancer over time. In Asian populations, optimizing the advantages of LDCT lung cancer screening while mitigating the risks of overdiagnosis and overmanagement is essential. Key strategies include predictive risk models for smokers and nonsmokers, implementing gender-specific screening approaches, providing education and training for healthcare professionals, and fostering shared decision-making, especially when considering active surveillance or surgery for early-stage lung cancer when pure ground-glass nodules are present. These measures are pivotal to the effectiveness of lung cancer screening programs in Asia.

### Supplementary Information

Supplementary Material 1.

## Data Availability

The datasets of the current study are available from the corresponding author upon reasonable request.

## Abbreviation

LDCT: Low-dose computed tomography
