# Supplementary figures and images for: Impact of annual trend volume of low-dose computed tomography for lung cancer screening on overdiagnosis, overmanagement, and gender disparities
Source: Cancer Imaging. 2024 Jun 12;24:73. doi: 10.1186/s40644-024-00716-5 (PMC11170916; doi:10.1186/s40644-024-00716-5)

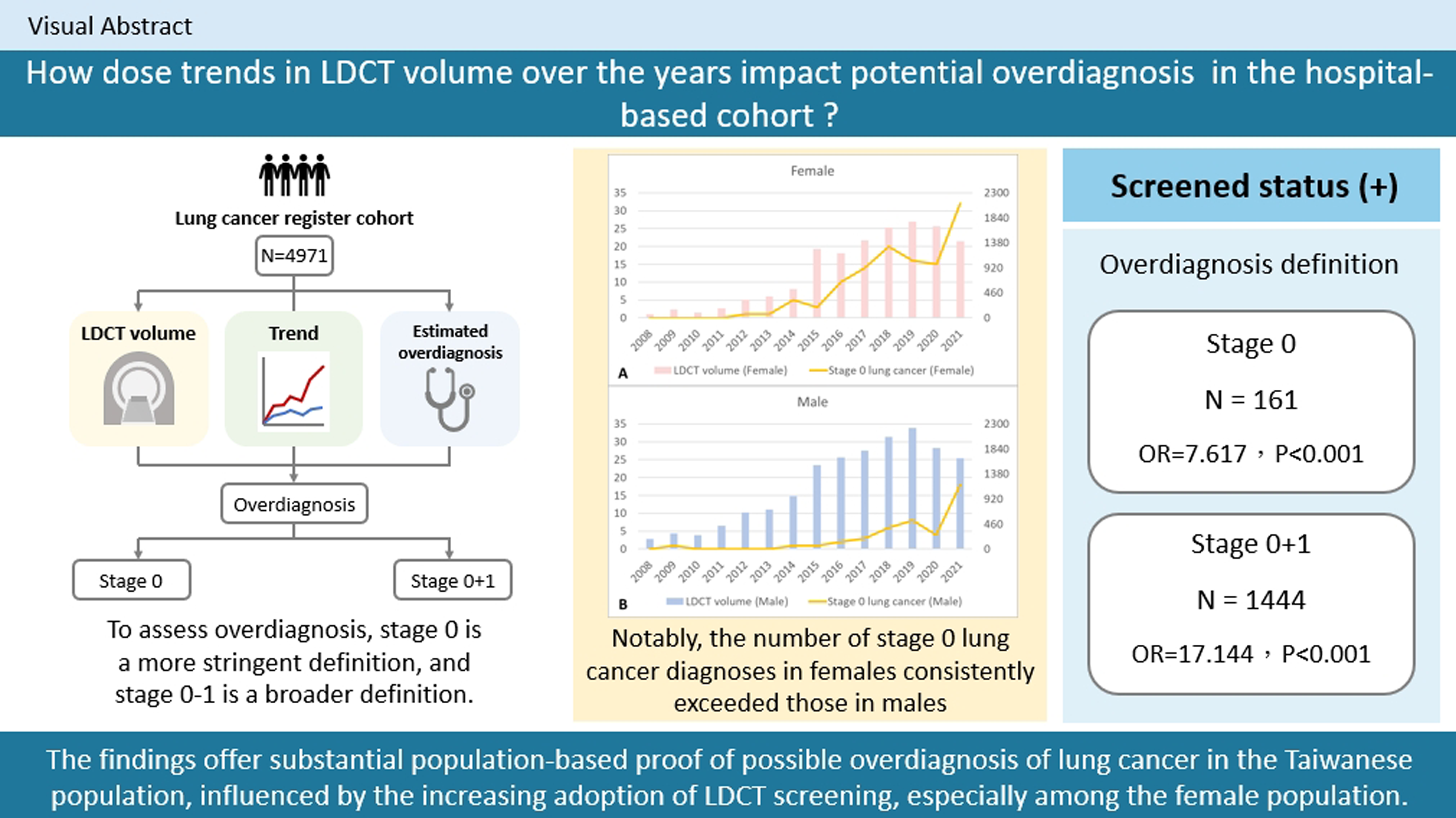

Supplement: Supplementary file 1 — Supplementary Material 1. [file 40644_2024_716_MOESM1_ESM.tif]
